# A Low-Power CMOS Microfluidic Pump Based on Travelling-Wave Electroosmosis for Diluted Serum Pumping

**DOI:** 10.1038/s41598-019-51464-7

**Published:** 2019-10-15

**Authors:** Pei-Wen Yen, Shiang-Chi Lin, Yi-Chun Huang, Yu-Jie Huang, Yi-Chung Tung, Shey-Shi Lu, Chih-Ting Lin

**Affiliations:** 1 Graduate Institute of Biomedical Electronics and Bioinformatics, National Taiwan University, Taipei, 10617 Taiwan; 2 Graduate Institute of Electronics Engineering, National Taiwan University, Taipei, 10617 Taiwan; 3 Research Center for Applied Sciences, Academia Sinica, Taipei, 11529 Taiwan

**Keywords:** Biomedical engineering, Electrical and electronic engineering

## Abstract

Microfluidic pump is an essential component in lab-on-chip applications. It is of importance to develop an active microfluidic pump with low-power and low-cost characteristics for portable and miniaturized diagnostic systems. Taking advantages of CMOS technologies, in this work, we report a low-power microfluidic pump based on travelling-wave electroosmosis (TWEO). Utilizing an integrated driving circuit, this monolithic CMOS microfluidic pump can be operated at 1.5 V driving voltage with a power consumption of 1.74 mW. The integrated driving circuit consist of a resistor-capacitor (RC) oscillator, a 90-degrees phase-shift square wave generator, and buffer amplifiers. Moreover, capabilities of the developed CMOS TWEO pump to drive diluted human serum are characterized. The flow rate of diluted human serum with dilution ratio of 1:1000 can achieve 51 μm/s. This is the first time demonstrating an *in-situ* CMOS-based microfluidic pump to drive the clinical diluted serum sample. As a consequence, this work demonstrates an essential component of CMOS biotechnologies for potential applications of portable *in vitro* diagnosis (IVD) systems.

## Introduction

Microfluidic devices play an important role in personalized diagnostic tools, which have been one of major trends in lab-chip developments^1^. Among various personalized platforms, mobile systems have become promising carriers for next generation personalized diagnostic tools. However, microfluidic devices lack of a low-power and miniaturized active driving mechanism to fit in mobile platforms. To conquer these barriers, nowadays, a number of researches work on different kinds of micropumps, including micromechanical pump^2^, electrowetting^3^, and electroosmosis^4^. Although most of these works demonstrated great potentials to drive microfluidics, few of them could be directly integrated with mobile systems that require low-power and low-voltage driving with simple manual operations. Therefore, it is important to develop a microfluidic pumping device, which is able to be integrated with electronics and circuits, for personal-mobile diagnostic platforms.

Harnessing advantages of integrated electronics, complementary-metal-oxide-semiconductor (CMOS) compatible technologies offer numerous benefits, such as system integrity, low cost, miniaturization, and mass production^5^. With abundant advantages aforementioned, CMOS technologies are appreciated to develop as biosensors for personal diagnostic platform. Therefore, various CMOS integrated sensing devices, e.g. microcantilevers^6^, ion-sensitive field-effect-transistors (ISFET)^7^, electrochemical impedance spectroscopy^8,9^, capacitance^10^, and silicon nanowire (SiNW)^11^, have been successfully developed and employed in clinical related biomolecular sensing. These CMOS biosensing technologies demonstrate great potentials to be used in personalized diagnostic tools. On the other hand, it is intriguing to implement a microfluidic pump by CMOS technique to monolithically build a chip platform for biomolecular diagnosis, including sample driving and detection. Among various microfluidic pumping techniques^12^, the electrokinetic fluid actuation offers an adequate method coupled with other bio-diagnostic components in a CMOS-based biomolecular diagnosis chip platform, especially with electrically operated sensors. To drive microfluidics by electrokinetic effects, electroosmosis is one of the major mechanisms^13^. However, most DC electroosmotic pumps require high voltage, e.g. tens of volts, and extra experimental setups. To conquer these problems, Cahill *et al*. designed a method to induce travelling-wave electroosmosis (TWEO) on a symmetry electrode array^14^. By using this AC electroosmotic phenomenon, the major concern of electrochemical reactions in electrokinetic mechanisms can be reduced because of its low-voltage driving. Thus, TWEO with simple electrode structure in linear array provides a potential candidate to perform an on-chip fluid actuation.

To realize a low-power on-chip microfluidic actuation, in this work, we have successfully developed an on-chip CMOS-based TWEO microfluidic pump driven with a monolithically integrated low-power circuit. By employing standard 0.35 μm CMOS Bio-MEMS processes with a post-deposited gold layer for biocompatibilities, this on-chip CMOS-based TWEO pump can be implemented with advantages of miniaturization and cost-effective manufacturing. This developed CMOS-based microfluidic pump has been experimentally demonstrated to have microfluidic actuations with 1.5 V driving voltage and tunable driving frequency. Based on experimental results, the total power consumption is measured below 1.74 mW while operating at 1.5 V driving voltage and 3k Hz driving frequency. Moreover, the developed CMOS pump demonstrates an ability to drive diluted serum samples by a designed digitalized integrated circuit generating travelling square waveform. For the first time, an integrated CMOS microfluidic pump is performed as a key element to actuate a phantom bio-fluidic sample. Therefore, this work not only develops a CMOS microfluidic pump but also promotes the development of CMOS based biodiagnostic system-on-chip for personalized diagnostics.

## Results

### Design principle

**Figure 1:**
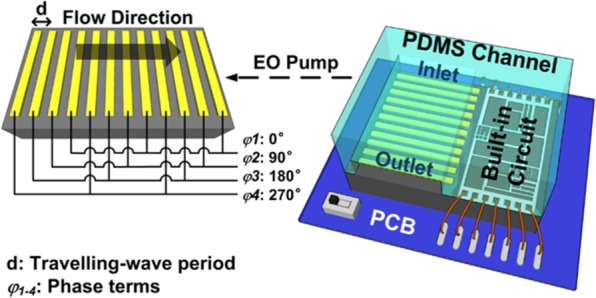
A schematic of the developed monolithically integrated CMOS TWEO microfluidic chip, and the inset is the enlarged schematic of pumping region.

In the developed CMOS TWEO microfluidic chip as schematically shown in Fig. 1, the on-chip electrode array consists of 60 units. Each unit has 4 consecutive electrodes to induce TWEO flow with equal phase shift in electrical potential by an integrated driving source. To be monolithically integrated in standard CMOS circuit, the driving signal has a voltage limitation of 3 V. To avoid surface topography issue from CMOS fabrication and interference from the travelling-wave electrical fields of the TWEO, the built-in TWEO driving circuits are placed away from the consecutive driving electrode array. To demonstrate the functionality of the developed CMOS microfluidic pump, finally, the CMOS TWEO chip is wire bonded on a print circuit board (PCB) and covered by a polydimethylsiloxane (PDMS) microfluidic channel.

To simulate the flow velocity of the developed CMOS TWEO micropump, we utilize the numerical simulation software, COMSOL Multiphysics (v4.3a, COMSOL, Stockholm, Sweden), to evaluate the performance of the TWEO flow. To simplify the simulation structure, in addition, we only consider one unit electrode array and set the flow-in and the flow-out boundaries as periodic conditions for flow field. The geometry parameters of the electrode array are set depending on the fabricated limitation of the 0.35 μm standard CMOS Bio-MEMS process.

The TWEO flow velocity is related to the voltage drop across electrical double layers, and the velocity calculation can be shown as^11^

1 $$\langle {u}_{x}\rangle =-\frac{\varepsilon }{4\eta }\cdot \frac{{C}_{s}}{{C}_{s}+{C}_{d}}\cdot \frac{\partial }{\partial x}[(\psi -{V}_{j}){(\psi -{V}_{j})}^{\ast }],$$

where *ε* is the liquid permittivity, *η* is the liquid viscosity, *indicates complex conjugate, *Ψ* is the electrical potential, *V*_*j*_ is the applied voltage on the electrode, *C*_*d*_ is the capacitance of the diffuse layers, and *C*_*s*_ is the capacitance of the stern layers. For the single mode wave, the expression of the applied voltage is shown as:

2 $${V}_{j}={V}_{0}{e}^{i(\omega t-{k}_{0}x)}.$$

Utilizing the calculated flow velocity, the flow field in the microfluidic channel can be calculated by treating the electrical double layer as a sliding wall boundary. By assuming no-slip conditions, the average pumping rate can be obtained as:

3 $${u}_{EO}=\frac{1}{L}{\int }_{-L/2}^{L/2}{u}_{x}dx.$$

**Figure 2:**
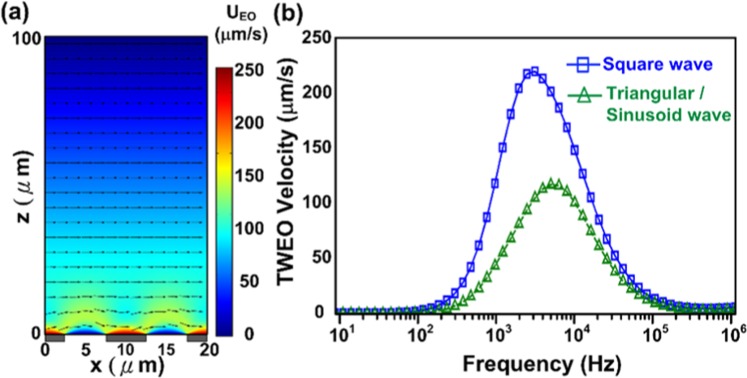
The numerical simulation results of the developed CMOS TWEO microfluidic pump. (**a**) The distribution of the TWEO flow velocity; (**b**) The TWEO pumping velocity as a function of the applied frequency with different driving waveforms.

Based on the previous boundary conditions, we are able to evaluate the TWEO velocity distribution shown in Fig. 2(a). Obviously, because the TWEO pumping source is resulted from the sliding electrical double layer, the flow velocity profile is nearly similar to the coquette flow whose velocity linearly decreases along the channel height. Thus, we could predict the fluid flow induced by the pump chip is non-uniform in vertical direction.

In previous works, the driving signal of TWEO devices is a sinusoidal waveform generated by an external function generator or multi-function data acquisition systems^15^. To be monolithically integrated with driving circuits, a perfect sinusoidal waveform generator with precisely 90-degreeds phase-shift outputs is considered as a power-hungry and area-consumed design. As a consequence, the on-chip TWEO driving circuit is re-examined by different driving waveforms, e.g. square, sinusoidal and triangular waveform. According to the Fourier transform, the square wave is decomposed as a superposition of sinusoidal wave with a series of frequencies, ω, 2ω, 3ω, 2(p + 1)ω. Therefore, the expression of the applied voltage is further shown as Eq. (4):

4 $${V}_{j}={\sum }_{p=0}^{\infty }{V}_{0}{e}^{i[(2p+1)\omega t-{k}_{0}x]}.$$

The Eq. (4) is implemented in the COMSOL simulation as the TWEO driving source, and the Eqs (1) and (3) are considered as the sliding velocity on electrode boundary condition. Figure 2(b) shows a numerical simulation of pumping velocity as a function of the applied frequency with different driving waveforms. Based on Fig. 2(b), the maximum flow velocity induced by square-wave signal is larger than the other one induced by sinusoidal wave, i.e. square-wave: 225μm/s, sinusoidal-wave: 115μm/s. Furthermore, the effects of triangular and sinusoidal simulation result are exactly consistent. To be implemented in CMOS integrated circuit, in addition, a square waveform driving circuit has advantages of low power consumption, easy implementation, and precise phase-shifting. Therefore, the square wave is an optimal choice for the TWEO driving signal due to its high flow velocity and easy implementation. It should be noted that the simulation results shown in Fig. 2(b) is obtained on the top of gold electrode surface, e.g. the sliding layer with the maximum flow velocity. On the other hand, the experimental velocity is obtained by the particle velocity, which is flowing with a lower flowing velocity at nearly middle-height of the microfluidic channel as shown in Fig. 2(a).

### Architecture of on-chip driving circuit

**Figure 3:**
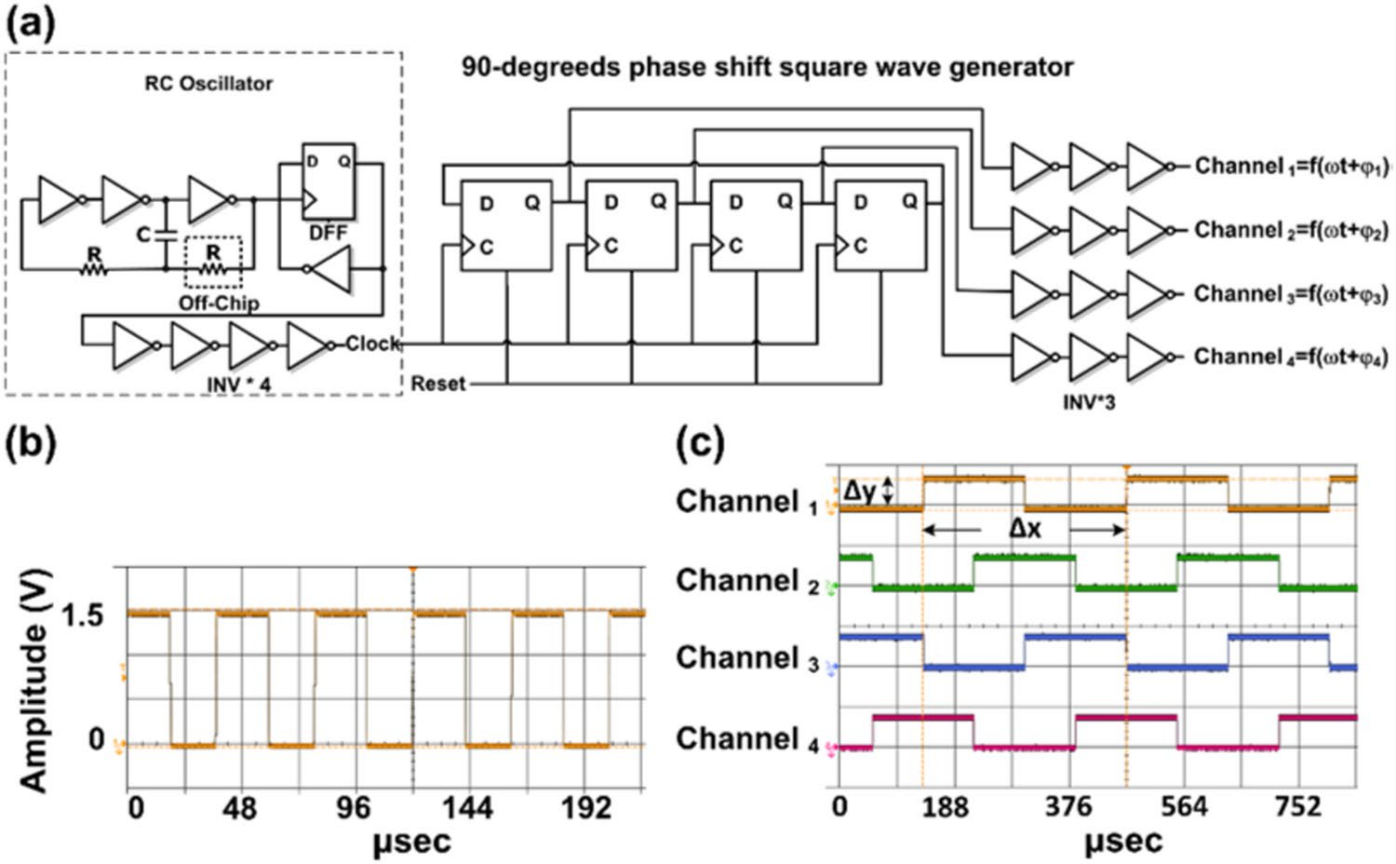
The travelling-wave built-in driving circuit. (**a**) The schematic architecture of implemented travelling-wave built-in driving circuit. (**b**) The tuned clock frequency of built-in ring oscillator clock is operated at 24.08 kHz. (**c**) The experimental output of 90-degreeds phase shift waveform measured under designed clock frequency. Δx and Δy are represented a period and amplitude of the output frequency of channel 1 with phase term is 0°, 1/Δx is measured as 2.985 kHz and Δy is the 1.5 V peak to peak voltage of the on-chip circuit driving output.

The schematic architecture of the travelling-wave driving circuit is showed in Fig. 3(a). This built-in circuit is designed to drive TWEO with 90-degrees phase-shift square wave generator. Where output signals are represented as ${\rm{f}}({\rm{\omega }}t+{{\rm{\phi }}}_{1-4})$, ${\rm{\omega }}t$ is the driving frequency and ${{\rm{\phi }}}_{1-4}$ are the phase delayed terms. According to the previous simulation result (Fig. 2(b)), the square wave has better driving capability to achieve maximum velocity than the sinusoid wave does. On the other hand, implementing a sinusoid-wave generator has problems of expensive, complicated, power consuming, and waveform distortion. For instance, the classical design of a multi-phase shift sinusoid-wave generator requires a modulating square-wave voltage-controlled oscillator (VCO) of variable frequency, a multi-phase generator, and square-to-sine wave converters. These converters are used to consecutively transform waveforms from square to triangle and triangle to sinusoidal shapes^16,17^. Based on these factors, therefore, the built-in driving circuit is designed based on a phase shift square-waveform generator to perform a stable and tunable driving ability. In brief, this built-in waveform generating circuit is composed of a clock generator, 90-degrees phase shift square wave generator, and a set of inverter buffer chains. The 90-degree phase shift square-wave signals implemented in digitized circuit and controlled by a clock generator. In this work, the clock generator is realized by a ring oscillator with its output connected to a D-Flip-Flop (DFF). In this schematic, the ring oscillator is composed by three invertors, an off-chip resistor, and a capacitor. The output frequency of the ring oscillator can be fine-tuned by adjusting an off-chip resistance value to change the RC frequency. Compare with other circuitry designs, this digitalized controlled square wave has an advantage of low power consumption, which is below hundreds microwatt. Furthermore, this digitally controlled square wave generator can be easily implemented and modulated by a clock generator with precisely phase shift terms and tunable output frequency.

To obtain a precise and tunable TWEO driving signal, the TWEO frequency is designed as one-eighth of the clock frequency. To improve the driving capability, in addition, a series inverter buffer chain is necessary to have a good driving waveform without distortions induced by the tunable TWEO loading. Because the consecutive electrode array are immersed and operated under liquid environment, the output loading of the phase shift square wave generator comes from the capacitance between the metal electrodes and the ionic liquid. These output loadings are determined by the terms of *C*_*d*_ and *C*_*s*_ in Eq. (1) from fluidic ionic liquid. In this work, in brief, Total output loading for four phase shift equal to 21.76 nF. Our driving buffer was designed for 10 nF/ phase shift. The total driving capability should be near to 40 nF.

#### Characterization of the design driving circuit

After the fabrication of CMOS chip, the on-chip electroosmotic pump device was wire bonded on the PCB and covered with PDMS microfluidic structure including protection of bonding pads. The built-in driving circuit is firstly validated to examine the driving capability. According to previous TWEO simulation result, the digitized travelling square waveform generator circuit is designed to operate nearly 3k Hz to achieve the travelling square wave maximum driving ability. As shown in Fig. 3(b), the clock frequency of ring oscillator clock is tuned and operated at 24.08k Hz, which is 8 times of TWEO operation frequency. Under this clock frequency, the four channels square wave output frequency can be generated with 90-degreeds phase shift sequentially. Figure 3(c) shows the experimental output of 90-degreeds phase shift waveform. Where channel_1–4_ represent the output waveform of 90-degreeds phase shift square wave signals with amplitude 1.5 V and frequency 2.985 kHz. One thing should be noted that the optimized driving frequency is different according to the ionic strength of driving solutions. In following experiments of on-chip electroosmotic pumping validation in ionic buffers and diluted serums, therefore, the travelling wave output driving frequency is set as nearly 3 kHz and 2.5 kHz respectively. In addition, the supplied voltage is set to have 1.5 V peak to peak output amplitude. In square wave driving, this setup can obtain the maximum flow velocity without strong electrolysis of the electrodes in the implemented design.

**Figure 4:**
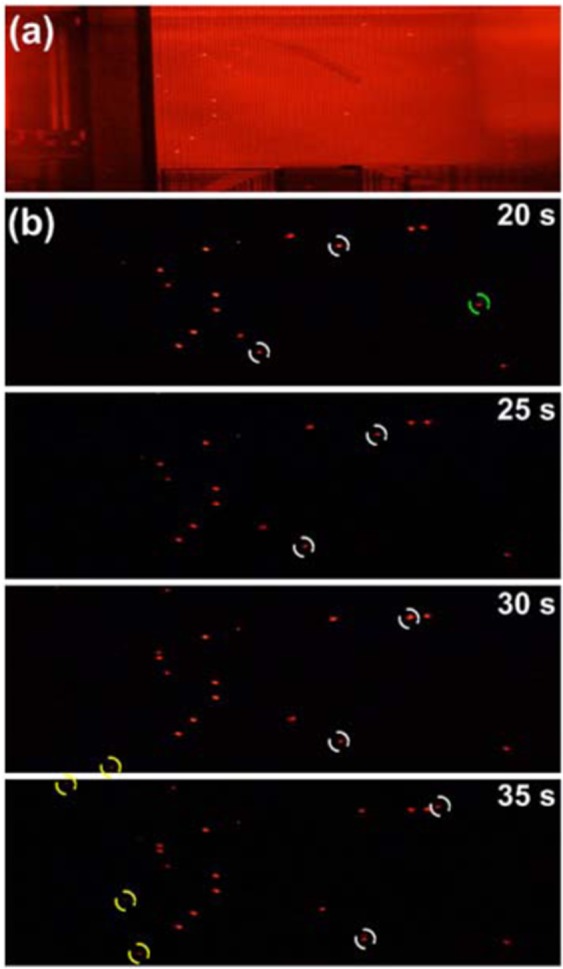
(**a**) The snapshot of the monolithically integrated CMOS TWEO microfluidic chip with bright field illumination. (**b**) The on-chip fluid pumping of diluted serum samples in time sequence.

To validate the implemented on-chip electroosmotic pump by an ionic buffer, we used 100 μM KCL with 1μm fluorescence beads as demonstration. Figure 4(a) shows the snapshot of device with bright field illumination and the Fig. 4(b) demonstrates the on-chip fluid pumping phenomenon in a time sequence manner. To observe the particle motion clearly, the bright field illumination is turned off during the videotaping period. The observation window of Fig. 4(b) kept the same as the Fig. 4(a). To clearly show the fluid pumping phenomenon, the particles are marked with various-colored circles. The white circles indicate the particles moving along the channel during the observing period. The green circles represent the particles moving out the outlet and the yellow circles mean the particles dragged from the inlet. The motions of these particles imply that the TWEO successfully actuated the fluid without interruption and discontinuity.

## Discussion

The TWEO flow is similar to the couette flow, so the flow velocity decreases linearly from the sliding boundary on the electrode to the non-slip channel top. The maximum velocity of particles is experimental examined as 33.85 μm/s. The flow velocity measurement result is obtained by moving particles near the electrode surface. In addition, the total driving power consumption of the experiment is 1.74 mW at 1.5 V driving voltage. It can be observed that the experimental maximum pumping velocity is smaller than the simulation results. The possible reasons of the slow fluidic velocity might result from the limitation of the Bio-MEMS fabrication process and the corrosion surface of the gold electrodes. In this chip, the width of the consecutive electrode is designed at the limitation of the Bio-MEMS fabrication process to increase the pumping velocity. Nevertheless, the fabrication limitation causes the non-uniform electrode width. This affects the uniformity and continuity of the TWEO flow. Furthermore, the corrosion layer, e.g. gold oxide on the top of gold electrodes, causes extra capacitance effect between the electrode and solution. To estimate this effect, a simulated flow velocity as a function of the gold-oxide-layer thickness on the top of gold electrodes can be demonstrated (as shown in Fig. S1 in Supplementary Materials). This result is derived from an additional capacitor in series connection with the electric double layer capacitor in the Randles circuit model. Additionally, a flow velocity distribution along z-direction with different oxide thickness can also be demonstrated (as shown in Fig. S2 in Supplementary Materials). According to these simulated flow velocity profiles, the suspended particles are not able to reach the highest velocity as the simulation results. It should be noted that the surface corrosion of gold electrodes also limits the life time of the on-chip TWEO microfluidic pump^18^. Although the life time of this on-chip pump device is limited, the implemented device is appropriate to be developed as a disposable mobile device for personalized applications because of considerations of epidemical contaminations and cross infectious.

To further demonstrate the capability of the fluid pumping chip in clinical applications, we took the human serum as the actuated liquid. Theoretically, the charging of the aqueous system is analogous to the charging of a capacitance and a resistance in series. The capacitance resulted from the electrical double layer, ${{\rm{\varepsilon }}}_{l}/{{\rm{\lambda }}}_{EDL}$, where ${{\rm{\varepsilon }}}_{l}$ is the liquid permittivity, and the ${{\rm{\lambda }}}_{EDL}$ is the thickness of Debye layer. The resistance is generated from the ohmic resistance of liquid, $L/{\rm{\sigma }}$, where *L* is the typical length of the aqueous system, and the ${\rm{\sigma }}$ is the conductivity of liquid. Thus, the charging time is of the order of RC time constant (τ_c_) shown as the Eq. (5)^19^. When the applied frequency is too high at the order of ${{\rm{\omega }}{\rm{\tau }}}_{c}\gg 1$, the most voltage drop across the bulk solution, so the induced charge in electric double layer is too small to generate the TWEO flow. On the contrary, the applied frequency is too low at the order of ${{\rm{\omega }}{\rm{\tau }}}_{c}\ll 1$, and then the voltage drop is mainly across the electric double layer which causes the electric field predominantly in vertical direction instead of the tangential direction. Thus, the frequency of maximum velocity occurs at the order of $1/{{\rm{\tau }}}_{c}$.

5 $${\tau }_{c}=\frac{\varepsilon }{{\lambda }_{EDL}}\frac{L}{\sigma }$$

Therefore, the optimal frequency to achieve maximum flow velocity is dependent on the sample conductivity, permittivity and thickness of Debye layer. Thus, it is clear that the driving frequency needs to be re-examined to attain the highest TWEO flow velocity for pure human serum. This is because the conductivity of human serum (S/m) is about three orders larger than the previous sample of 100 μM KCL solution (mS/m). To verify the conductivity influence on TWEO flow velocity, the pump chip was employed with a series of human serum diluted with various v/v ratio, i.e. 1:1000, 1:100, and 1:10. As Table 1 shows, the TWEO flow velocities decreases with the increment of the conductivity of diluted serum. The reason is that the high conductivity, under the applied frequency of 2.5 kHz, induces the strong screening effect of electrical double layer. In contrast, the serum diluted by one thousand times is able to achieve the higher flow velocity than other ones, which corresponds the results of 100 μM KCL solution. Therefore, the results demonstrate the importance of frequency design for various sample conductivity. We further show the averaged electoosmotic velocity as a function of the solution conductivity (as shown in Fig. S3 in Supplementary Materials). In addition, it should be addressed that we elevated the electrical field by 10 μm pitches design of whose electrode width and gap are both 5 μm^18,20^. To optimize the pitch, we should further derive the electroosmotic slip velocity. It clearly shows that the maximum electroosmosis slip velocity is proportional to the wavenumber of the traveling wave signal. Therefore, it is able to increase the maximum TWEO velocity with shrinking the electrode pitch. However, the TWEO working frequency will be shifted to higher values.

**Table 1:** The summary table illustrates the measured osmotic flow velocity and conductivity of diluted serums with different dilution ratio.

Serum Dilution Ratio	1000	100	10
Conductance (mS/m)	1.6 ± 0.1	16.7 ± 0.2	163.5 ± 1
Flow Velocity (μm/s) @2.5 kHz, V_pp_ = 1.5 V	51.75	9.96	~0

Based on above experiments, we have successfully demonstrated the microfluidic actuation with diluted human serum sample by a fully integrated on-chip electroosmostic pump system. Moreover, this on-chip pump device is able to behave as a fluidic driving platform with good drivability which is able to integrate with various CMOS based biosensors as a complete bio-diagnosis system-on-chip (SoC). Compared with previous works^21,22^, the driving voltage and process feasibility is the major advantages. It should be noted that the trade-off is the flow rate, which is not outstanding compared with the other micropumping mechanism. From systematic aspects, on the other hand, CMOS-based sensing devices (e.g. silicon nanowire FET) has been demonstrated to good detection limit and sensitivity. To integrate with these sensors, some high concentration biomolecules, such as glycated hemoglobin and insulin^23^, would easily saturate these sensors. Under this detection limitation, as a consequence, the serum samples should be diluted thousand times by buffer to fit in the sensitivity of the ultrasensitive devices. Therefore, the proposed on-chip pump has potential to be integrated with CMOS-based ultrasensitive biosensor as bio-diagnosis SoC for personal diagnostics application.

In conclusion, we have demonstrated a low-power TWEO microfluidic pump which is driven by monolithically built-in CMOS circuits. Utilizing TWEO driving mechanism with linear consecutive electrode array, the developed micropump is able to be miniaturized and operated at 1.5 V driving voltage. To drive the TWEO micropump, a monolithically integrated tunable 90-degreeds phase shift square wave generator is used to achieve the driving signal and optimize the driving frequency. This is the first time demonstrating an *in-situ* CMOS-based microfluidic pump to drive the clinical diluted serum sample with the flow velocity nearly 50 μm/s. The total power consumption of the developed TWEO microfluidic pump is measured as 1.74 mW. This work shows a promising capability of a CMOS-based on-chip TWEO microfluidic pump providing a steady flow field and velocity in clinical sample liquid environments. Because of standard CMOS fabrication process, the developed fluidic manipulating device can be easily implemented with various CMOS-based biosensing devices to accomplish a miniaturized personal *in vitro* diagnosis (IVD) system. As a consequence, this work demonstrates an essential component of CMOS biotechnologies for potential applications in mobile healthcare systems.

## Methods

### Implementation of on-chip microfluidic pump

**Figure 5:**
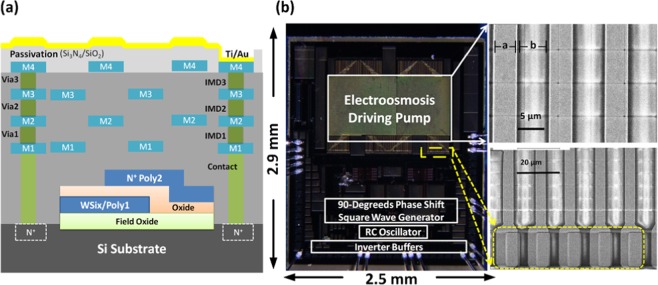
(**a)** The schematic diagram of the contents layers of 0.35 μm CMOS Bio-MEMS process. In this schematic, metal layers (M_1_-M_4_) are the layers for electronic circuit layout routing, Via_1_-Via_3_ are the interconnection between adjacent metal layers, and inter-metal dielectric layers (IMD_1_-IMD_3_) are the dielectric layers used to electrically insulate interconnect lines. (**b**) The images of the fabricated chip and the SEM picture of the fabricated electrodes and interconnections array. The yellow dash square represents the double –size width designed of contact points between Ti/Au and M_4_ layers.

The developed CMOS microfluidic pump is realized by CMOS Bio-MEMS process. This process is composed of a standard 0.35μm two-polysilicon and four-metal layers (2P4M) CMOS process and a post-deposited gold (Au) layer on the top of the chip by post-CMOS micromachining process. As shown in Fig. 5(a), a standard multilayer structure can be deposited, patterned, and fabricated based on the 2P4M CMOS process. This structure can be used to implement functional circuits. After circuit fabrications, a Ti/Au (30/300 nm) layer is deposited on the top of the CMOS passivation layer, i.e. oxide/nitride, to form the consecutive electrode array for TWEO microfluidic pump. It should be noted that it is important to maintain the surface-topography integrity of deposited Ti/Au electrodes because the electrical field distribution can be affected by the surface roughness of the electrodes. To reduce the surface roughness of electrode structures, stacked dummy layers are designed by two staggered metal layer setting with tetra-ethoxysilane (TEOS) inter-metal dielectric layers. One of the staggered dummy layer sets is composed by metal layer 1(M1) and metal layer 3(M3), and the other is metal layer 2(M2) and metal layer 4(M4). Utilizing the staggered dummy layer structure, the surface roughness induced by Ti/Au deposition can be minimized to nearly 0.35 μm. Compared with the size of microfluidic channel, i.e. with channel height and width are nearly 200 μm and 0.8 mm respectively, the surface roughness can be neglected and treated as a planar boundary. Figure 5(b) shows the photograph of the fabricated chip and the SEM picture of the fabricated linear electrode array. To design the linear electrode array, it has two constrains: (1) the maximum flow velocity; (2) the post-CMOS process limitations. Since the flow velocity is proportional to the tangential electric field on the electrode and the fabrication-resolution constrain of Ti/Au layer, the period of the electrode array is designed as 10 μm, i.e. electrode and gap widths are both 5 μm. Furthermore, the electrical connections between driving circuits and electrodes are achieved by directly contact of Ti/Au layer and PAD (Metal 4, M4) layer. To ensure the contact between Ti/Au and M4 layers, the Ti/Au contact-point width should be double-size as that of M4 contact-point. This can avoid the deposited-layer discontinuity results from the height difference induced by fabrication processes.

The implemented CMOS TWEO microfluidic pump is composed of a linearly consecutive electrode array, a 90-degreeds phase-shift travelling wave built-in driving circuit, and a PDMS microfluidic channel. The fabricated chip size is 7.5 mm^2^ with 1.5 V supplied voltage. Since the measurement of the on-chip fluidic pump is in aqueous solution, the bonding pad and PCB is passivated by PDMS to avoid leakages from contact to liquid buffer by short-circuit effect. To accomplish this on-chip CMOS microfluidic pump, the entire consecutive parallel electrode array pumping area is covered by a PDMS microfluidic channel fabricated by soft-lithography.

### On-chip circuit validation

For the built-in driving circuit validation, the developed on-chip electroosmotic pump device is wire bonded on the designed printed circuit board (PCB). During the experimental operation, the driving voltage power is supplied by E3631A triple output power supply (Agilent Technologies, Santa Clara, CA). And the travelling square wave output are measured and recorded by a DSO-X 3034A digital storage oscilloscope (Agilent Technologies, Santa Clara, CA).

### Sample preparations

In this work, the fluorescent stereo microscope (Leica M165C, Leica Microsystems, Wetzlar, Germany) coupled with a digital single-lens reflex camera was used to record particle movements. To suppress the travelling wave dielectrophoresis, the red fluorescent beads of 1μm (F-13082, Invitrogen, Carlsbad, CA) were employed to explore the TWEO flow field on the chip. These fluorescent particles were soaked in 100 μM KCl solution (P5405, Sigma-Aldrich Co., St. Louis, MO) as our actuated ionic sample. To investigate the practical capability of the pumping chip, we further employ the human blood serum as our actuated sample. The pure human blood was previously centrifuged to get rid of all the blood cells. (IRB No: FEMH-IRB-101091-F). The purified serum was diluted by different ratio of deionized water (DI) solutions to investigate the feasibility of real biosamples.

## Supplementary information

Supplementary Information
