# Supplementary material for: A Low-Power CMOS Microfluidic Pump Based on Travelling-Wave Electroosmosis for Diluted Serum Pumping
Source: Sci Rep. 2019 Oct 15;9:14794. doi: 10.1038/s41598-019-51464-7 (PMC6794323; doi:10.1038/s41598-019-51464-7)
Supplement: Supplementary file 1 — Supplementary Information [file 41598_2019_51464_MOESM1_ESM.docx]

**A Low-Power CMOS Microfluidic Pump Based on Travelling-Wave Electroosmosis for Diluted Serum Pumping**

# Pei-Wen Yen‡^a^, Shiang-Chi Lin‡^b^, Yi-Chun Huang^a^, Yu-Jie Huang^b^, Yi-Chung Tung^c^, Shey-Shi Lu^b^, and Chih-Ting Lin^a,b*^

^a^ Graduate Institute of Biomedical Electronics and Bioinformatics, National Taiwan University, Taipei 10617, Taiwan

^b^ Graduate Institute of Electronics Engineering, National Taiwan University, Taipei 10617, Taiwan. Fax: +886 2 2368 1679; Tel: +886 2 3366 9603; E-mail: [timlin@ntu.edu.tw](mailto:timlin@ntu.edu.tw)

^c^ Research Canter for Applied Sciences, Academia Sinica, Taipei 11529, Taiwan.

**Supplementary Information**

Eqn. (S1) shows the normal current conservation which is boundary condition on the electrode. To take into account the effect of oxide layer, the effective capacitance term turns into the series capacitance of gold oxide layer and electrical double layer shown as eqn. (S2)

$\sigma\frac{\partial\psi}{\partial z}=i\omega C_{eff}(\psi-V_{i})$ (S1)

$\frac{1}{C_{eff}}=\frac{1}{C_{EDL}}+\frac{1}{C_{oxide}}$ (S2)


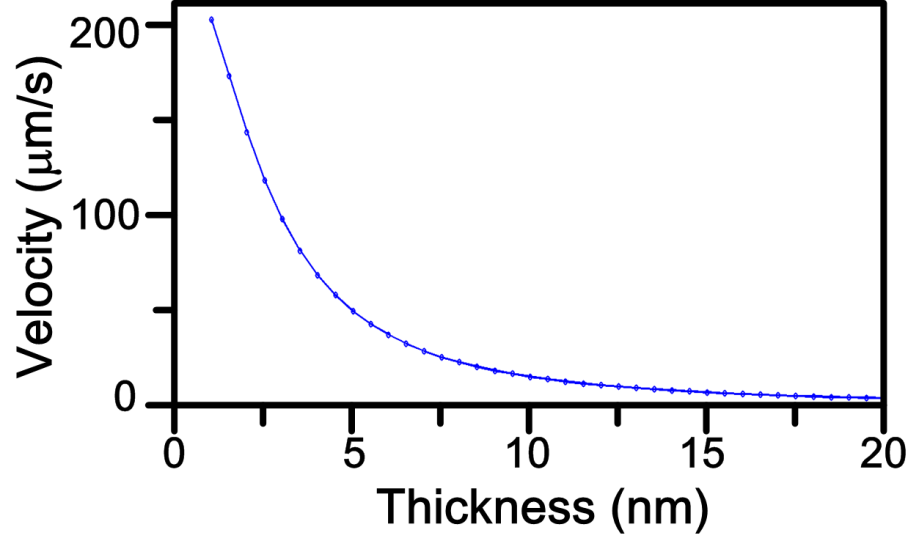


Figure S1 The averaged electroosmotic velocity corresponding to various thicknesses of gold oxide layers.


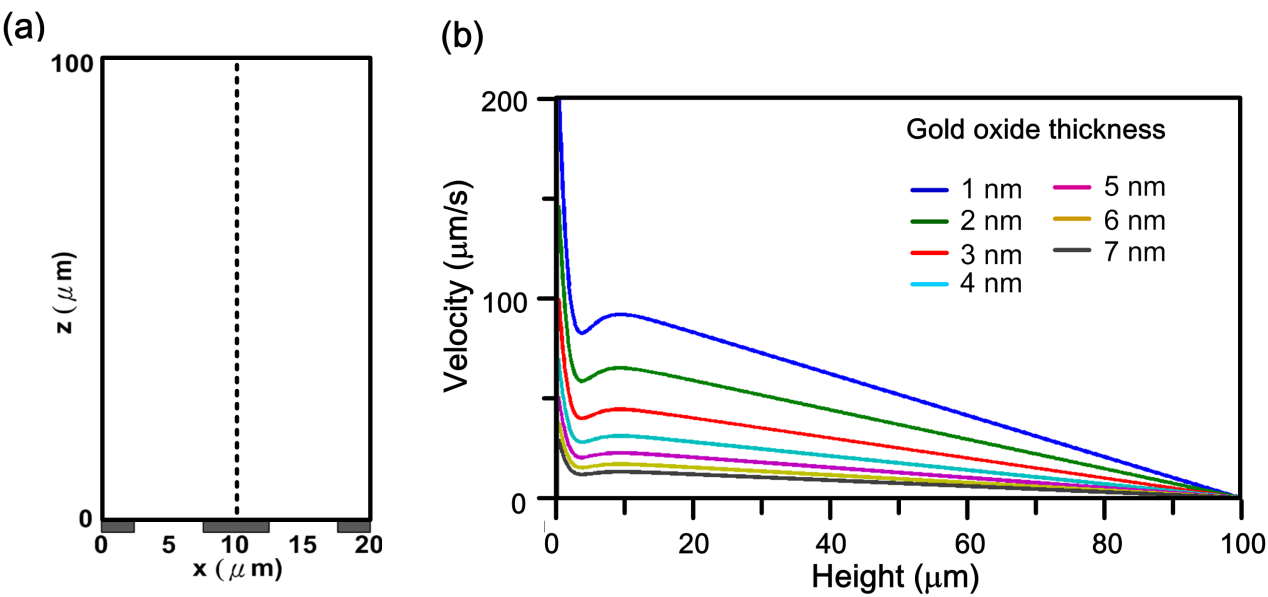


Figure S2 (a) The simulation structure for the various thicknesses of gold oxide, and the broken line above the middle electrode is the analysis line. (b) The electroosmotic velocity distribution as function of heights in channel with various thicknesses of gold oxide layers.


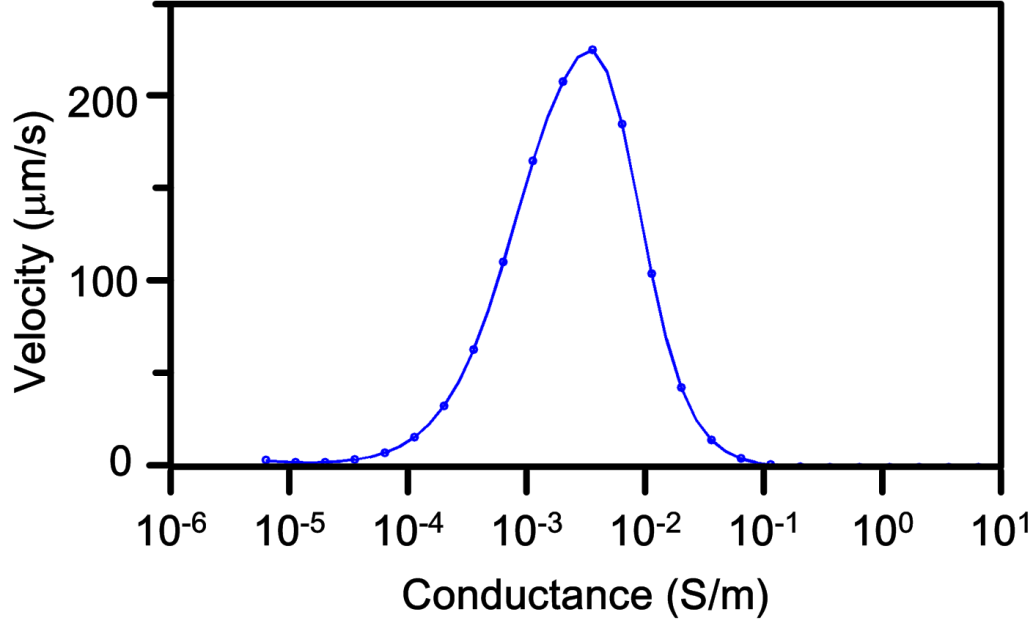


Figure S3 The averaged electroosmotic velocity corresponding to various conductance of solution with the applied frequency 3000 Hz.
